# Effect of fermented blueberry on the oxidative stability and volatile molecule profiles of emulsion-type sausage during refrigerated storage

**DOI:** 10.5713/ajas.19.0094

**Published:** 2019-05-28

**Authors:** Hengyue Zhou, Xinbo Zhuang, Changyu Zhou, Daming Ding, Chunbao Li, Yun Bai, Guanghong Zhou

**Affiliations:** 1 Key Laboratory of Meat Processing and Quality Control, MOE; Key Laboratory of Meat Processing, MOA; Jiangsu Synergetic Innovation Center of Meat Processing and Quality Control; College of Food Science and Technology, Nanjing Agricultural University, Nanjing, 210095, China

**Keywords:** Emulsion-type Sausage, Antioxidant, Volatile Profile, Refrigerated Storage

## Abstract

**Objective:**

The aim of this work was to assess the effect of fermented blueberry (FB; 2%, 4%, and 6%) on the oxidative stability and volatile molecule profiles of emulsion-type sausage stored at 4°C for 28 days.

**Methods:**

The antioxidant activity of FB was determined through radical-scavenging activity against 2, 2-diphenyl-1-picrylhydrazyl (DPPH) and hydroxyl radicals. Four formulations of sausage treatments with different FB levels (0%, 2%, 4%, 6%) were prepared, then peroxide value (POVs), thiobarbituric acid-reactive substances (TBARS) values, protein carbonyls and thiol groups were measured. The aroma profiles of sausages for each treatment was also determined.

**Results:**

The half maximal inhibitory concentration indicated that FB had greater scavenging ability than ascorbic acid against DPPH and hydroxyl radicals. Sausages with FB significantly retarded increases in POVs and TBARS, as well as in the content of protein carbonyls during all storage days (p<0.05). Particularly, 4% and 6% FB-treated sausages had better oxidation inhibition effects. However, FB accelerated the reduction in thiol groups (p<0.05). Additionally, FB inhibits the excessive formation of aldehyde compounds; for example, hexanal, which may cause rancid flavors, decreased from 58.25% to 19.41%. FB also created 6 alcohols (i.e., 2-methyl-1-propanol, 3-methyl-1-butanol, and phenylethyl alcohol), 5 ester compounds (i.e., ethyl acetate, ethyl lactate, and ethyl hexanoate) and 3-hydroxy-2-butanone in the sausages that contribute to sausage flavors. The principal component analysis showed that the aroma profiles of sausages with and without FB are easily identified.

**Conclusion:**

The addition of FB could significantly reduce the lipid and protein oxidation and improve oxidative stability for storage. Also, adding FB could inhibit rancid flavors and contribute to sausage flavors.

## INTRODUCTION

Emulsion-type sausages are prepared with lean meat and fat, then chopped, cased and finally subjected to thermal processing under controlled temperature and relative humidity [1]. However, the heating treatment and storage period can accelerate lipid oxidation, which is a major cause of off-flavors and a greasy taste in meat products [2]. Excessive lipid oxidation can severely diminish the product acceptability for consumers [3]. Protein oxidation occurs simultaneously with lipid oxidation in meat systems, and protein oxidation also has detrimental effects on meat quality, including tenderness, water-holding capacity and nutritional quality [4]. Therefore, there is increasing interest in control of the oxidation in meat products by using the antioxidant agents from synthetic and natural sources [5]. However, excessive intake of the synthetic antioxidants reportedly cause cancer and deformities [6]. As a result, natural sources are of interest as substitutes to synthetic antioxidants. The application of spices, vegetables, fruit extracts and wine industry residues have received the most attention by consumers and meat processors [7].

Currently, consumers prefer sausages with good flavors and nutritional value. Fermented juice, vegetable juice, and extracts derived from berries have been added to sausages to satisfy different customers and the market demand for product diversification [8]. In a typical Italian product (*Salama da sugo*) up to 15% (v/w) grape ferment is added to the meat to form a crucial aromatic profile, such as isoamyl alcohol and phenylethyl alcohol, The relative percentage of esters are already high immediately after stuffing due to the wine [9]. Feng et al [1] produced frankfurter-type sausage with red wine, they found that adding 5% wine could increase the a* value, improve the texture and also introduced new volatiles (alcohol and ester compounds) to the frankfurters.

Blueberry are rich in phenolic compounds (particularly anthocyanins, caffeic, chlorogenic, *p*-coumaric acid, and ferulic acid) with high antioxidant potential, the flavonols (predominantly quercetin derivatives) and proanthocyanidins are also preaent [10,11]. Fermented blueberry (FB), which fermented by probiotics (yeast and lactic acid bacteria) in the proper temperature and pH for at least one month, will improve the antioxidant ability of blueberry and increase aroma compounds [11,12]. Jiang [13] showed that 2, 2-diphenyl-1-picrylhydrazyl (DPPH) scavenging ability and hydroxyl radical scavenging ability of FB increased by 3.6% and 2.4% respectively after fermenting 64 days. Su and Chien [14] observed that acids produced by fermentation can inhibit “sweat” odor of FB. And the most important aroma-active compounds of FB were low olfactory thresholds such as acetic acid, 2/3-methyl-butanoic acid, phenethyl acetate, and 2-phenylethanol. As a result, the addition of FB can affect the flavor profile and inhibit oxidation of lipids and proteins.

We previously demonstrated that the addition of FB can improve the sensory evaluation and color of frankfurters, but decrease the hardness and cohesiveness of sausages and with no significant effect on the textural springiness. And the results of electronic nose showed that the treatments added with FB were significantly different from the control (p<0.05). On this basis, this study was conducted to evaluate the effect of adding FB on the oxidation and aroma profile of emulsion-type sausage during storage.

## MATERIALS AND METHODS

### Materials

The FB, which formation was liquid and fermented by yeast and lactic acid bacteria for two months, was obtained from Tiansui Biotechnology Company (Shandong, China). Fresh pork hams and back-fat were obtained from a local commercial processor (Yurun Group, Jiangsu, China). The chemicals used for a scavenging assay (e.g. DPPH and ascorbic acid) and lipid oxidation (e.g. 2-Thiobarbituric acid [TBA]) were analytical purity. The rest of the ingredients, such as sodium phosphate (Sushi Group, Jiangsu, China) and casings (Yurun, China), were used for sausage production.

### Preparation of fermented blueberry and an antioxidant activity assay

The FB was centrifuged at 10,000 g for 10 min at 4°C and the supernatant removed for determination [13].

#### DPPH radical scavenging activity of FB

The DPPH radical scavenging activity was determined according to Jia et al [15]. Two mL of a 20 mg/mL DPPH solution (dissolved in 95% ethanol) was thoroughly mixed with FB from 5 μg/mL to 25 μg/mL and ascorbic acid aqueous solutions from 20 μg/mL to 100 μg/mL (2 mL), respectively. Ascorbic acid was chosen as a positive control in the present study as it has a strong ability to scavenge free radicals [16]. The mixture was incubated in the dark for 30 min, and the corresponding absorbance (A_1_) and absorbance of the samples mixed with ethanol (A_2_) at 517 nm was read. In addition, the absorbance (A_0_) of the control (DPPH solution without samples) at 517 nm was also recorded. The scavenging rate was calculated by the following equation:

$$\text{Scavenging rate of DPPH radical }(\%)=(1-({\text{A}}_{1}-{\text{A}}_{2})/{\text{A}}_{0})\times 100$$

#### Hydroxyl radical scavenging activity of FB

The method described by Xu et al [16] was used to measure the hydroxyl radical scavenging activity. Volumes of 0.6 mL of 6 mM salicylic acid-ethanol solution, 2 mL of 6 mM FeSO_4_·7H_2_O aqueous solution, and 1.4 mL of 6 mM H_2_O_2_ were mixed with 2 mL of different concentrations of FB and ascorbic acid. The mixture was vortexed and then incubated in a water bath at 37°C for 30 min. The absorbance of the mixture with (A_1_) or without salicylic acid (A_2_) was recorded at 532 nm. Additionally, the absorbance of the control without samples (A_0_) at 532 nm was determined. The scavenging rate was obtained using the same equation with DPPH.

### Sausage manufacture

**Figure 1:**
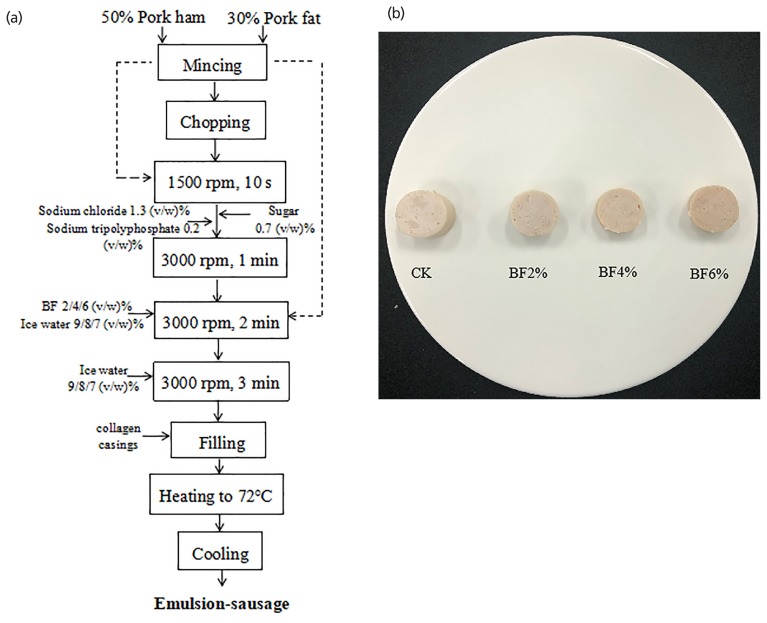
Fermented blueberry (FB); schematics illustrating the amount and type of ingredient added to manufacture emulsion-sausages (a); Photographic image of the emulsion-sausages (b).

All sausages were made with the same materials and ingredients including: pork meat (50%), pork back-fat (30%), water (20%); and ingredients including sodium chloride (1.3%), sodium tripolyphosphate (0.2%), sugar (0.7%), and test contents that differed by treatment. Four formulations of sausage treatments with different FB levels (0%, 2%, 4%, 6%) were prepared. The first meat batter serving as the control (CK) was prepared with 20% ice water. The other meat batter serving as treatment 2 (2% FB), treatment 3 (4% FB), and treatment 4 (6% FB) was prepared with 18%, 16%, and 14% ice water, respectively. The calculation method of the additives and preparation procedure referred to the document of Pil-Nam et al [17] and with some modifications. After chopping, the meat batter was immediately stuffed into 28-mm diameter collagen casings with a vacuum stuffer (SV-3, Hakka Brothers Machinery Co., Ltd., Sichuan, China) and linked every 200 mm. Finally, the sausages were cooked with steam until the internal temperature reached 72°C. After cooking, the sausages were immediately soaked in cold water to cool and then the water was drained. The schematic of the production process and the photographic image of the emulsion-type sausages were shown in Figure 1a–1b, respectively. The sausages were vacuum packaged (DC800-FB-E, Promarksvac Corporation, Ontario, CA, USA) in polyethylene bags (nylon/polyethylene, 9.3 mL O_2_/m_2_/24 h at 0°C) and finally assigned into 3 different storage periods; 1, 14, and 28 days and kept at 4°C.

### Determination of peroxide values

The peroxide values (POVs) were determined according to Xu et al [16]. Specifically, 5 g of the lipid extract was dissolved with 50 mL of a glacial acetic acid and isooctane mixture (v/v = 3:2). The mixture was shaken vigorously to achieve full dissolution of the lipid extract. Then, 0.5 mL of saturated potassium iodide solution was added to the mixture, which was left undisturbed in the dark for 3 min. Subsequently, 30 mL of distilled water was added, followed by incubation in the dark. Titrated 0.01 mol/L of the sodium thiosulfate solution into the mixture until the blue faded. The POV (mmol/kg) was determined by the following equation:

P=0.5×1,000×ΔV×C/M

ΔV is the volume of the consumed sodium thiosulfate solu tion (mL), C is the concentration of sodium thiosulfate solution (mol/L), and M is the mass of the examined lipid extract (g).

### Determination of thiobarbituric acid-reactive substances values

The thiobarbituric acid-reactive substances (TBARS) value was measured by the method of Zhang et al [18] with slight modification. Briefly, 5 g of minced sausage was homogenized with 10 mL distilled water for two periods of 30 s. Then, 0.2 mL of the sausage was homogeneously mixed with 0.2 mL of 8.1% sodium dodecyl sulphate, 1.5 mL of a 0.8% (w/v) TBA solution, 1.5 mL of 20% acetate buffer (pH 3.5), and 0.6 mL distilled water under vigorous shaking. The samples were incubated at 95°C for 1 h in a water bath (ZKSY-600, Keer, Nanjing, China). After cooling, 1 mL of distilled water and 4 mL of n-butanol-pyridine solution (butanol/pyridine:15/1) were added, and the samples were vortexed (MX-F, SCILOGEX, Shanghai, China) for 30 s. After centrifuging for 10 min at 4,000 g, the color of the butanol-pyridine layer was measured at 532 nm by a spectrophotometer (SpectraMax M2e, Molecular Devices Corporation, Silicon Valley, CA, USA). Results were expressed as milligrams of malondialdehyde (MDA) per kilogram meat sample (mg/kg) according to a standard curve of MDA.

### Determination of protein carbonyls

The protein carbonyl content was evaluated following the method of Jongberg et al [19]. Briefly, carbonyl groups were reacted with 2,4-dinitrophenylhydrazine to develop protein hydrazones. The reaction products were detected by measuring the absorbance at 370 nm in a spectrophotometer (SpectraMax M2e, Molecular Devices Corporation, USA), while protein concentrations were calculated by measuring the absorbance at 280 nm and using bovine serum albumin (BSA) as a standard. The carbonyl group content was expressed as nmol carbonyl/mg protein using an extinction coefficient of 21.0 mM^−1^×cm^−1^.

### Determination of protein thiol groups

The thiol content was measured using 5,5-dithiobis (2-nitrobenzoic acid) according to the method described by Zhang et al [18]. The protein thiol concentration was calculated using a standard curve prepared from a 1 mM L-cysteine stock solution. Protein concentrations were determined using a BSA standard curve. The results were expressed as nmol thiol/mg protein.

### Volatile compounds

Volatile compounds of the five different batches of fermented sausages and FB were monitored from casings after 28 days using gas-chromatographic-mass spectrometry coupled with solid phase microextraction (GC-MS-SPME). The method was according to Benet et al [20]. Samples (5 g) were placed in 20 mL sterilized vials, sealed by a poly tetra fluoro ethylene/silicon septa and heated for 10 min at 45°C. Volatiles were adsorbed for 40 min on a fused silica fiber covered by 50/30 μm carboxen polydimethyl siloxane (CAR/PDMS StableFlex; Sigma Aldridge Corporation, Shanghai, China). Adsorbed molecules were desorbed in the gas chromatograph for 3 min at 250°C. Separation of volatile flavors was performed using a DB-WAX capillary column (50 m×320 μm×1.2 μm) column.

The conditions were as follows: injection temperature, 250°C; detector temperature, 200°C; carrier gas (He) flow rate, 1 mL/min. The oven temperature was programmed as follows: 40°C for 3 min; from 40°C to 70°C, at 3°C/min; from 70°C to 230°C, at 10°C/min, then holding for 8 min. The ionization potential of MS was 70 eV, and the scan range was 33 to 450 *m/z*. Volatile compounds were quantified by the peak area. Additionally, linear retention indices (RI) of all identified volatile were calculated as the retention time of the volatile normalized to the retention times of adjacently eluting n-alkanes, and compared to reported RI contained in the Agilent Hewlett-Packard NIST 98 and Wiley 8.0 to further support the identifications. The GC–SPME results were expressed as the mean of three replications.

### Statistics analysis

Three replications of samples were used for each analysis. Data were analyzed by one-way analysis of variance procedure of SAS (SAS 9.1 version, Carly, NC, USA) for different treatments. The differences in the mean values were compared by Duncan’s multiple comparison method (p<0.05) and presented as standard error (mean±SE). With regard to the aroma profile, principal component analysis (PCA) was carried out with Statistica 7.0 (StatSoft Italia srl, Vigonza, Italy). Two principal components, factor 1 and factor 2 were retained to determine treatment scores.

## RESULTS AND DISCUSSION

### Scavenging effect of fermented blueberry on DPPH radicals

**Figure 2:**
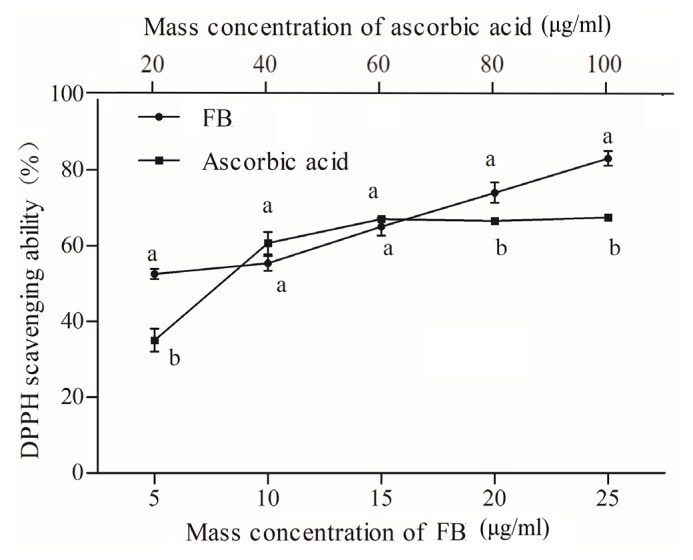
Fermented blueberry (FB); scavenging activity of FB and ascorbic acid against DPPH; error bars refer to the standard error obtained from triplicate sample analysis. Different letters in the same concentration indicate significant difference (p<0.05).

The FB has been proven to have potent antioxidant activity [21]. Figure 2 shows the scavenging rate of FB with an increase in concentration. The half maximal inhibitory concentration (IC_50_) for FB and ascorbic acid were 2.961 μg/mL and 32.390 μg/mL, respectively, implying that FB had greater scavenging ability than ascorbic acid against DPPH. As the concentration increased, the DPPH scavenging rate of FB raised faster. When the concentration was from 60 to 100 μg/mL, the scavenging ability of ascorbic acid gradually stabilized, which was less than FB due to the auto-oxidation of ascorbic acid, resulting in no increase in clearance. Oh et al [12] indicated that the radical scavenging capacity of FB juice is positively correlated with phenolic content. They also found that probiotic-mediated FB showed higher DPPH free radical scavenging activity than the unfermented control samples.

### Scavenging effect of blueberry on hydroxyl radicals

**Figure 3:**
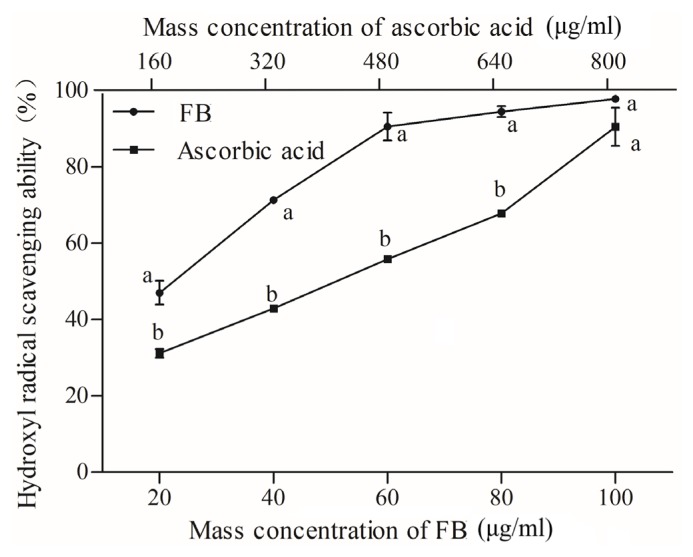
Fermented blueberry (FB); scavenging activity of FB and ascorbic acid against hydroxyl radical; error bars refer to the standard error obtained from triplicate sample analysis. Different letters in the same concentration indicate significant difference (p<0.05).

The hydroxyl radical, as the most reactive and powerful oxidizing active oxygen, is able to directly react with lipids and its primary oxidation products (ROOH, hydroperoxy-alkyl hydroperoxide). FB and ascorbic acid had IC_50_ values of 21.073 μg/mL and 423.01 μg/mL. Thus, FB had higher scavenging activity than the reference ascorbic acid standard (Figure 3). The highest inhibitory effects of the 100 μg/mL FB was 97.85%, which was not significantly different than 800 μg/mL ascorbic acid. In addition, FB increased with their concentration in the reaction mixture. Therefore, FB can be considered as an excellent radical scavenger at high concentrations. The high radical scavenging activity of FB might be contributing by containing abundant phenolic material. Additionally, FB as an antioxidant is believed to intercept the free radical chain of oxidation and to produce hydrogen from the phenolic hydroxyl groups, thereby forming a stable end product [22].

### Peroxide values

The POVs are intermediate hydroperoxides of fat oxidation, commonly used in meat products to indicate the degree of fat oxidation. As shown in Table 1, the high value of the POVs means that massive intermediate products of lipid oxidation accumulates [23]. The POVs of the control and samples supplemented with 2% FB increased with the range of 0 to 14 days and then remained steady. However, the treatments with 4% and 6% FB remained steady throughout the storage time at about 0.61±0.05 mmol/kg. During the storage period, the POVs of the batters treated with FB were significantly (p<0.05) less than that of the control sample. On day 14, POVs for the control reached peak value (1.13 mmol/kg) while the values for the sausages treated with FB continued to range from 0.61 to 0.66 mmol/kg. These results indicated that addition of FB can significantly inhibit the generation of hydroperoxides in the sausage.

During the initial stage of oxidation, the formation rate of hydro peroxides exceeds the decomposition rate, but this reversed during the later stages [24]. So in the later storage of period the POVs of all treatments keep stability. The addition of antioxidants could clear the formation of hydro peroxides, which result in the low POVs in the treated samples. Xu et al [16] also found that emulsion-type sausage produced hydroperoxides reduced slightly during longer storage. The POVs change over time, which is a dynamic two-stage process.

### Thiobarbituric acid-reactive substances values

The TBARS values is expressed in mg MDA/kg sample, the values indicate the amount of the secondary oxidation product of fat [25]. Overall, the TBARS values of sausages increased over the 28 days in all sausage samples, indicating that lipid oxidation occurred during refrigerated time (Table 1). The FB-treated sausages showed significantly lower TBARS values (p<0.05) than the control on each of the sampling days. In addition, the control increased from 1.75 mg MDA/kg to 2.73 mg MDA/kg during all storage times (p<0.05), while the FB treatments were always below 1 mg/kg. Significant differences (p<0.05) were also found between the addition of 2% and 6% FB treatments on 28 day; the greater the addition, the lower the TBARS value. The results showed that the addition of FB at all levels retarded lipid oxidation in the emulsion-type sausage.

Blueberries are a great source of bioactive compounds such as polyphenols (i.e., phenolic acids, flavonols, anthocyanins, tannins) and ascorbic acid [26]. They may act as strong antioxidants, able to decrease the incidence of oxidative stress damage. Berry extracts usually as antioxidants are incorporated in meats as water soluble and water insoluble extracts and powders [27]. Jia et al [15] found that adding black currant significantly decreased TBARS values 75% to 92% compared to the control and the inhibition effect was positively corrected with the amount of addition. This results is similar to the inhibition effect of FB in the experiment. Muzolf-Panek et al [7] also suggested that blueberries can protect meatloaf against oxidation during 12 days of storage due to values of bioactive compounds in blueberries, like 2.84 to 3.09 μg/g dry weight resveratrol, which belongs to the group of stilbenes. They also detected 205.4 to 841.3 μg/g dry weight quercetin of flavonols and phenolic acids, such as caffeic, ferulic and *p*-coumaric acids. The accumulation of MDA in muscle foods involves a straight loss of quality as most of these compounds contribute to the deterioration of color and flavor of meat products [2]. The FB has excellent DPPH radical scavengers and hydroxyl radical scavengers, so it could block a radical chain reaction in the lipid oxidation. The phenolic compounds in FB have the potential to donate hydrogen atoms to radical species, and oxidized to a phenoxyl radical themselves. The phenoxyl radicals are rather stable radicals and could prevent further initiation of lipid or protein oxidation [28].

### Carbonyls content

Carbonyl compounds are mainly produced by four pathways: direct oxidation of amino acid side chains such as arginine and histidine [29]; cleavage of peptide backbone; reaction with reducing sugars; binding of non-protein carbonyl compounds [30,31]. Therefore, carbonyl content is one of the most general parameters to evaluate protein oxidation [32]. The amount of protein carbonyls on the control batch and sample treated with 2% FB increased significantly (p<0.05) while the addition of 4% and 6% FB had no changes throughout days 1 and 14 as shown in Table 1. In addition, the control samples showed the highest values on each of the sampling days. Particularly, the protein oxidation inhibitory capacity ranged between different amounts of FB during later storage days from 28% to 39% and 29% to 35%, respectively. Thus, the addition of FB showed significant inhibitory effects on the production of protein carbonyls when compared to the control group (p<0.05).

Similarly, red wine is also a berry ferment. A provious study found that the addition of 5% and 10% (v/w) red wine to frankfurt sausages can significantly inhibit carbonyl generation during sausage processing and the inhibition ability is significantly greater than celery powder [1]. The phenolic compounds in blueberries might scavenge the reactive oxygen species and inhibit protein degradation in muscle foods during cooking or storage [33]. In addition, phenolic compounds prevent protein oxidation by acting as metal chelators and radical scavengers [34], by retarding lipid oxidative reactions, and binding to proteins to form complexes. However, the inhibitory effect was less intense than that in lipid oxidation. These results may be attributed to faster lipid oxidation than that of protein oxidation and to the likely covalent binding of phenolic compounds to protein that would mask their antioxidant activity on proteins [35].

### Thiol groups

Protein oxidation usually results in decreased levels of thiol groups which can be attributed to the formation of disulphide bonds by oxidation [36]. The thiol groups of all samples rapidly decreased compared to day 1 (p<0.05). On days 1 and 14, the control sample showed the highest value among four sausage treatments (Table 1). The results demonstrate that throughout days 1 and 14, the presence of FB in sausages caused an increase in the loss of thiol groups. However, there was no significance between the samples treated with 6% FB and the control at day 28 of storage.

Zhang et al [18] utilized sage to hinder the sausage oxida tion and found the similar phenomenon of loss of thiol groups. Similarly, Jongberg et al [19] revealed that the addition of white grape extract accelerated the loss of thiol groups, but reduced the formation of myosin cross-links in beef patties. They also explained that there could be interactions between thiol groups and the polyphenols in the extract. Ortho-phenolic structures are highly susceptible to adduct formation with nucleophilic thiols to form thiol-quinone adducts. Chlorogenic acids, one of the most important polyphenols in blueberries [37], is an ortho-phenolic compound that can interact with thiol to form adducts. The addition of FB to sausages resulted in faster loss of the thiol groups, which may due to the ortho-phenolic compounds react with the nucleophilic thiols. However, the phenomenon that thiol groups of the samples treated with 6% FB had no difference with the control in the later period of storage may relate to the phenolic compounds in high concentration of FB, which can scavenge free radicals and compete with thiol groups for trapping free radicals.

### Volatile profile

The volatile profile (expressed as relative percentage of the different compounds) of four experimental treatments and FB alone at the end of storage (28 days) is reported in Table 2. The main identified chemical families were alcohols, aldehydes, ketones, and esters. Several acids and furans were also identified. Such a profile is similar to previously described profiles in similar non-smoked products with no spices added [38].

Under these conditions, aldehydes represented the major ity of molecules detected and accounting for approximately 2/3 of the total volatile compound peak area in the absence of FB and about 1/3 to half with the FB addition. Aldehydes are the most prominent volatiles produced during lipid oxidation and have been used to successfully follow lipid oxidation in meat products. Among aldehydes, hexanal, nonanal, and pentanal were the most abundant volatiles detected in the four treatments, whereas octanal and n-heptanal showed intermediates values. Among these straight-chain saturated aldehydes, hexanal, which is formed from the oxidation of n-6 unsaturated fatty acids (i.e., linoleic acid), indicates muscle lipid oxidation was more effective than any other volatile compound [39]. Octanal and nonanal was mainly derived from oleic acid oxidation, octanal contributes to a fruity odor, and nonanal has an aroma of rose and orange [40]. These compounds are typically among the most abundant in emulsion sausages [38]. The presence of hexanal was responsible for about 58.25% of the total peak area in CK, 39.05% in 2% FB, 25.96% in 4% FB and 19.41% in 6% FB. The results showed that hexanal decreased significantly with FB addition (p<0.05). Hexanal, and generally aliphatic aldehydes, produce grassy, rancid, floral notes depending on their concentration [41], indicating that the addition of FB can inhibit the production of rancid flavors in sausages out to day 28. The conclusion was consistent with the TBARS values. Some other minor volatiles, such as trans-2-undecenal in treatment CK, was likely derived from the oxidative decomposition of polyunsaturated fatty acids. These have low olfactory thresholds and are also related to intense rancidity perception [42].

Five esters were found in FB-treated samples. This class of compounds is generally responsible for fruity and flowery notes that can contribute to sausage flavors [8]. Ethyl lactate was the major ester compound detected, which was possibly derived from the esterification of alcohols and acids because of the relatively low content FB [1]. Nine other esters, including ethyl citrate, diethyl succinate, and phenylacetate, present in FB were not observed in the emulsion-type sausages that included FB. While the dilution factor of adding wine to the frankfurters may be the cause, it is also possible as those compounds have relatively low boiling points (<120°C), they are likely to evaporate during thermal processing [1].

The total alcohol content increased after adding FB to the meat batters, which can cause some unique volatile flavors. Ethanol was the major alcohol compound in FB. However, ethanol was undetected with the addition of FB, which is unexpected and likely due to evaporation during processing. Another possibility was esterification of ethanols on day 28, which explains the 6 esters that appeared in treatments with FB [43]. These observations further indicated that some secondary reactions occurred at the processing steps [1]. In particular, 1-octen-3-ol, which imparts a strong mushroom flavor, was present in significantly higher percentages in the sample without FB [44]. Phenethylic alcohol, a stronger alcohol typically produced by yeast in FB [45], was detected in low amounts in the batches treated with FB.

Fewer ketones were detected in all the sausages. The per centage of 2,3-octanedione was significantly greater in the sausages without FB (p<0.05) because ketone is another major oxidation product of lipid. Most ketone compounds have a milky and fruity aroma. However, the higher threshold results in less contribution to flavor than aldehydes. 3-Hydroxy-2-butanone was only detected in the treatments with FB. This volatile compound is related to cheese and butter, which can contribute to aroma in sausages at low thresholds [46].

Acids, at the end of storage, mainly represented by acetic and hexanoic acids, were found at levels comparable to those in sausages with wine [45]. Acetic acid, octanoic acid, and decanoic acid mainly derived from FB, Su and Chien [14] also found those compounds in FB juice and vinegar. Furan compounds were mainly products of linoleic acid oxidation, 2-pentyl-furan has a vegetable-like aroma and earthy aroma, as well as a bean flavor [47]. The compound in samples treated with FB was significantly less than the control due to the addition of antioxidant.

The PCA is a mathematical procedure for resolving sets of data into orthogonal components, whose linear combinations (principal components) approximate the original data to any desired degree of accuracy. In most cases, two principal components were sufficient to explain a most of the variation in the original variables, thus resulting in a considerable compression in data. Combined with the data in Table 2, sausages at the end of storage were used for a PCA for better evidence of differences in the aroma profiles of sausages in relation to the FB added. The first two factors accounted for 89.10% of the total variability. Generally, the sum of factors exceeded 85% and was considered to represent the original data information. The first factor accounted for 67.89% of the variability and 21.21% by the second factor. The high value of variability associated with factor 1 is not surprising and can be explained by the compounds derived from the addition of FB.

**Figure 4:**
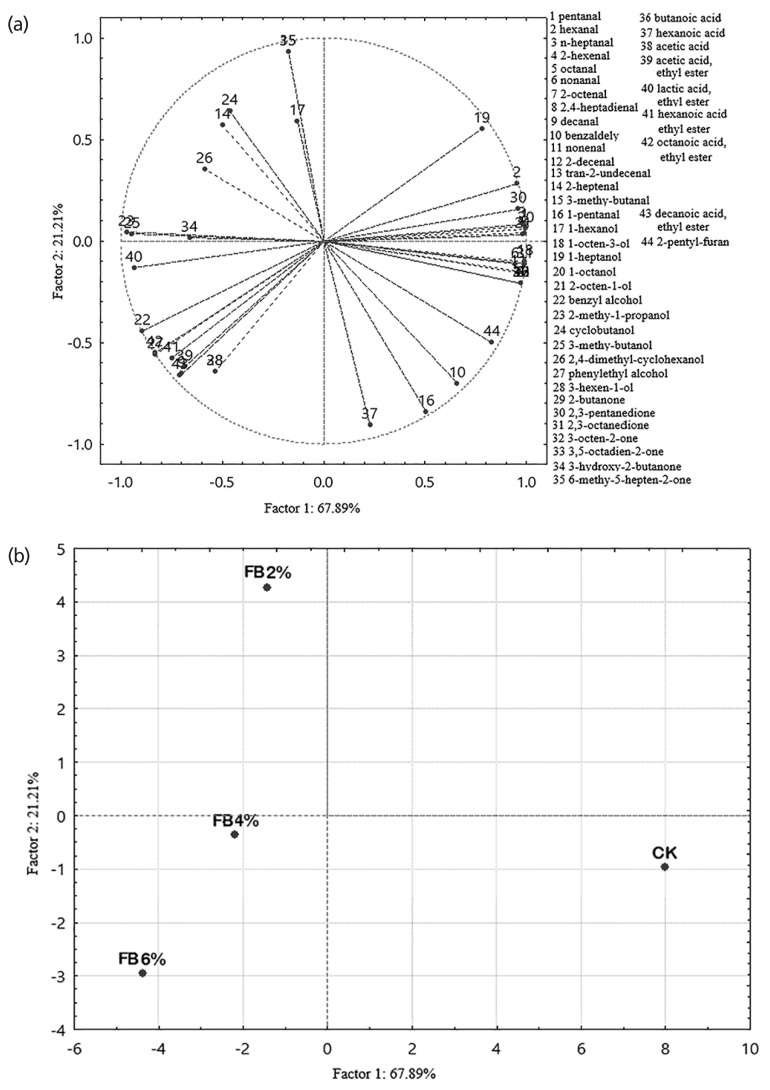
Fermented blueberry (FB); PCA loading plots of the volatile compounds selected on the first two factors (a) and PCA loading plots on the first two factors (b) obtained from the PCA of emulsion-sausages produced with different amounts of FB at the storage of 28 day. PCA, principal component analysis.

Figure 4a shows that for the first two factors, factor 1 was strongly related with most aldehydes detected (variables 1 to 9, 11 to 15) by lipid oxidation, as well as 2-pentyl-furan, 2,3-pentanedione and alcohols, i.e. 1-octanol (44, 30, 20, 18, 19). In contrast, a very negative correlation was also found with some alcohols and ethyl lactate (22, 23, 25, 40). The variability of factor 2 was very negatively correlated with hexanoic acid (37), 1-pentanal (16), and benzaldehyde (10) and, to a lesser extent, phenylethyl alcohol, 3-hexen-1-ol (27, 28) and other esters (39, 41, 42, 43). This factor was positively associated to 6-methyl-5-hepten-2-one (35), followed by four alcohols 1-hexanal, cyclobutanol, 1-heptanol, and 2,4-dimethyl-cyclohexanol (17, 24, 19, 26) and 2-heptenal (14). Figure 4b reports the PCA loading plots on the first two factors of the 4 sausages in relation to the amount of FB added. The sausages were easily separated into two groups (control and treatments with FB) along Factor 1 which were highly correlated with aldehydes, ethanol and esters. The FB addition would promote or inhibit these substances in the process and storage period, which was demonstrated above. Factor 2 was able to discriminate the treatments with different FB levels and was highly correlated with these substances belonging to FB itself.

## CONCLUSION

The FB has excellent DPPH and hydroxyl radical scavenging activities. The FB addition could effectively retard lipid oxidation in emulsion sausages as indicated by lower POVs and TBARS values, as well as protein oxidation. However, FB did accelerate the loss of thiol groupss in sausages during the storage due to ortho-phenolic structures. In the volatile molecule profiles, due to protection of unsaturated fatty acid from oxidative degradation, FB inhibits excessive formation of aldehydes compounds such as hexanal. The FB was also crucial for the aromatic profile of sausages, which can have a marked effect on product volatile molecule profiles. The PCA showed that the FB addition results in significantly different volatile molecule profiles between different sausages. In conclusion, FB as a natural ingredient has great potential in meat products to retard lipid and protein oxidation and alter the volatile molecule profiles.

## Figures and Tables

**Table 1:** Lipid oxidation and protein oxidation of emulsion-sausage with varying percentages of FB during refrigerated storage

Parameters	Storage day	Treatment1)			

		CK	FB2%	FB4%	FB6%
POVs (mmol/kg meat)	1	0.70±0.03^a^^C^	0.58±0.05^b^^B^	0.61±0.02^b^^A^	0.59±0.02^b^^A^
	14	1.13±0.11^a^^A^	0.66±0.02^b^^A^	0.61±0.02^b^^A^	0.61±0.01^b^^A^
	28	0.97±0.05^a^^B^	0.65±0.03^b^^A^	0.62±0.02^b^^A^	0.63±0.03^b^^A^
TBARS value (mg/kg meat)	1	1.75±0.04^a^^C^	0.41±0.23^c^^B^	0.51±0.03^bc^^B^	0.71±0.06^b^^A^
	14	2.25±0.07^a^^B^	0.74±0.08^b^^A^	0.74±0.07^b^^A^	0.72±0.11^b^^A^
	28	2.73±0.05^a^^A^	0.85±0.08^b^^A^	0.82±0.03^bc^^A^	0.74±0.02^c^^A^
Carbonyl contents (nmol/mg protein)	1	1.23±0.12^a^^B^	1.01±0.06^b^^B^	1.13±0.05^ab^^A^	1.06±0.11^ab^^A^
	14	1.81±0.13^a^^A^	1.31±0.14^b^^A^	1.18±0.11^b^^A^	1.11±0.10^b^^A^
	28	1.92±0.05^a^^A^	1.37±0.09^b^^A^	1.28±0.09^b^^A^	1.24±0.10^b^^A^
Thiol groups (nmol/mg protein)	1	251.47±2.39^a^^A^	232.83±7.25^ab^^A^	210.07±36.10^ab^^A^	200.03±30.98^b^^A^
	14	196.10±8.42^a^^B^	187.13±3.18^a^^B^	156.80±7.98^b^^A^	132.70±7.97^c^^B^
	28	198.37±19.88^a^^B^	139.13±14.29^b^^C^	155.10±33.66^ab^^A^	163.07±16.52^ab^^AB^

FB, fermented blueberry; POVs, peroxide values; TBARS, thiobarbituric acid-reactive substances.

1) CK, control, without fermented blueberry; FB2%, FB4%, FB6%, emulsion-type sausage incorporated with 2%, 4%, and 6% fermented blueberry.

a–c Values with different letters in the same row differ significantly (p<0.05).

A–C Values with different letters in the same column within a parameter differ significantly (p<0.05).

**Table 2:** Volatile compounds expressed as % of total peak area of emulsion-sausage with varying percentages of FB at the storage of 28 days

Compounds	RI	NIST	Treatment1)				

			FB	CK	FB2%	FB4%	FB6%
Aldehydes							
Pentanal	976	976	-2)	5.33±0.69^a^	1.63±0.37^b^	0.59±0.25^c^	-
Hexanal	1,077	1,078	-	58.25±1.23^a^	39.05±4.86^b^	25.96±5.23^c^	19.41±2.52^d^
N-heptanal	1,171	1,177	-	3.14±0.64^a^	1.51±0.81^b^	0.84±0.12^b^	0.78±0.28^b^
2-Hexenal	1,210	1,216	-	0.20±0.04	-	-	-
Octanal	1,278	1,277	-	1.45±0.74^a^	0.77±0.15^b^	0.78±0.23^b^	0.5±0.14^b^
Nonanal	1,380	1,380	-	4.63±0.29^a^	1.80±0.08^c^	2.71±1.01^b^	1.29±0.38^c^
2-Octenal	1,421	1,427	-	1.71±0.17^a^	0.38±0.09^b^	0.37±0.09^b^	0.29±0.03^b^
2,4-Heptadienal	1,488	1,503	-	0.11±0.03	-	-	-
Decanal	1,492	1,493	-	0.05±0.03^b^	0.07±0.04^b^	0.13±0.09^ab^	0.30±0.23^a^
Benzaldehyde	1,517	1,520	1.16±0.14	0.35±0.08^a^	0.17±0.01^c^	0.28±0.05^ab^	0.26±0.04^b^
Nonenal	1,531	-	-	0.51±0.22^a^	0.08^b^	-	0.06±0.01^b^
2-Decenal	1,641	1,636	-	0.32±0.09	-	-	-
Trans-2-undecenal	1,752	1,751	-	0.13±0.05	-	-	-
2-Heptenal	1,311	1,320	-	-	0.41±0.03^a^	-	0.24±0.13^a^
3-Methyl-butanal	914	916	-	-	0.06±0.01	-	-
2-Furan-carboxaldehyde	1,457	1,459	3.39±0.24	-	-	-	-
Alcohols							
Ethanol	947	947	28.89±2.81	-	-	-	-
1-Pentanol	1,259	1,258	-	2.03±0.23^a^	0.99±0.18^a^	1.39±1.29^a^	1.81±1.34^a^
1-Hexanol	1,357	1,356	-	0.48±0.05^a^	0.83±0.38^a^	1.07±1.17^a^	0.25±0.19^a^
1-Octen-3-ol	1,449	1,449	-	6.09±0.8^a^	1.45±0.21^b^	1.24±0.17^bc^	0.75±0.30^c^
1-Heptanol	1,456	1,456	-	0.52±0.15^a^	0.41±0.14^ab^	0.32±0.08^b^	-
1-Octanol	1,555	1,555	-	0.50±0.07^a^	0.22±0.06^b^	0.2±0.05^b^	0.1±0.01^c^
2-Octen-1-ol	1,611	1,616	-	0.39±0.06^a^	0.1±0.01^b^	-	-
Benzyl alcohol	1,878	1,878	-	0.13^b^	0.17±0.04^b^	0.22±0.04^a^	0.25±0.04^a^
2-Methyl-1-propanol	1,107	1,107	0.45±0.08	-	0.18±0.12^a^	0.14^a^	0.23±0.12^a^
Cyclobutanol	1,142	-	-	-	0.26±0.18^a^	-	0.13±0.14^a^
3-Methyl-1-butanol	1,222	1,223	2.54±0.31	-	1.68±0.78^a^	2.44±0.68^a^	2.02±0.01^a^
2,4-Dimethyl-cyclohexanol	1,541	-	-	-	0.14±0.03^a^	-	0.12±0.01^a^
Phenylethyl Alcohol	1,914	1,914	5.56±0.44	-	0.29±0.08^c^	0.94±0.15^b^	1.41±0.3^a^
3-Hexen-1-ol	1,390	-	-	-	-	-	0.09±0.02
Ketones							
2-Butanone	900	900	-	0.26±0.43	-	-	-
2,3-Pentanedione	1,056	1,056	-	0.13±0.02^a^	0.05±0.01^b^	-	-
2,3-Octanedione	1,315	1,325	-	6.67±0.37^a^	1.15±0.3^b^	0.96±0.11^b^	0.41±0.18^c^
3-Octen-2-one	1,400	1,414	-	0.2±0.06	-	-	-
3,5-Octadien-2-one	1,567	1,567	-	0.06±0.03	-	-	-
3-Hydroxy-2-butanone	1,296		-	-	0.15±0.07^a^	0.40±0.41^a^	0.16±0.11^a^
6-Methyl-5-hepten-2-one	1,329	1,329	-	-	0.05±0.05	-	-
Acids							
Butanoic acid	1,626	1,626	-	0.02	-	-	-
Hexanoic acid	1,843	1,844	-	0.38±0.04^a^	-	0.37±0.17^a^	0.35±0.02^a^
Acetic acid	1,441	1,441	4.13±0.27	-	-	-	1.41±0.81
Octanoic acid	2,056	2,056	0.60±0.02	-	-	-	-
Decanoic acid	2,272	2,272	0.73±0.01	-	-	-	-
Ester							
Acetic acid, ethyl ester	886	886	2.32±0.48	-	0.09±0.03^b^	0.2±0.05^b^	0.82±0.2^a^
Lactic acid, ethyl ester	943		2.56±0.01	-	29.34±5.39^b^	52.01±5.6^a^	49.50±19.41^a^
Hexanoic acid, ethyl ester	1,216	1,221	1.81±0.77	-	0.32±0.17^a^	0.59±0.02^a^	1.8±1.64^a^
Octanoic acid, ethyl ester	1,429	1,430	4.48±0.75	-	0.14±0.01^c^	0.36±0.06^b^	0.63±0.16^a^
Decanoic acid, ethyl ester	1,630	1,630	11.50±1.81	-	-	0.27±0.07^a^	0.32±0.19^a^
Nonanoic acid, ethyl ester	1,533	1,533	0.12±0.03	-	-	-	-
Succinic acid, diethyl ester	1,692	1,690	6.13±0.98	-	-	-	-
Benzeneacetic acid, ethyl ester	1,785	1,785	0.24±0.01	-	-	-	-
Dodecanoic acid, ethyl ester	1,844	1,843	9.55±3.53	-	-	-	-
Tetradecanoic acid,ethyl eater	2,049	-	0.94±0.44	-	-	-	-
Hexadecanoic acid, ethyl ester	2,256	2,256	3.99±1.45	-	-	-	-
Ethyl hydrogen succinate	2,381	2,395	0.65±0.50	-	-	-	-
Ethyl linoleate	2,530	2,532	0.56±0.24	-	-	-	-
Dibutyl phthalate	-	-	1.13±0.14	-	-	-	-
Other							
2-Pentyl-furan	1,207	1,213	-	0.7±0.17^a^	0.19±0.05^b^	0.23±0.05^b^	0.38±0.40^b^
Eugenol	2,170	2,171	0.17±0.04	-	-	-	-

FB, fermented blueberry; RI, retention indices; NIST, National Institute of Standards and Technology.

1) CK, control, without fermented blueberry; FB2%, FB4%, FB6%, emulsion-type sausage incorporated with 2%, 4%, and 6% fermented blueberry.

2) Not detected under the adopted conditions.

a–d Values with different letters in the same row differ significantly (p<0.05).
